# Supplementary material for: Modeling the stability of severe acute respiratory syndrome coronavirus 2 (SARS-CoV-2) on skin, currency, and clothing
Source: PLoS Negl Trop Dis. 2020 Nov 9;14(11):e0008831. doi: 10.1371/journal.pntd.0008831 (PMC7676723; doi:10.1371/journal.pntd.0008831)
Supplement: S1 Fig — (PDF) [file pntd.0008831.s001.pdf]

Supplementary Figure 1. Geometric mean ratio of half-lives, averaged across all three temperatures.

Effect	Temperatures Compared		Ratio of Half-Lives	p-value
Temperature	4°C	22°C	20.35	**<.0001**
Temperature	4°C	37°C	73.14	**<.0001**
Temperature	22°C	37°C	3.59	**<.0001**
