# Supplementary material for: Modeling the stability of severe acute respiratory syndrome coronavirus 2 (SARS-CoV-2) on skin, currency, and clothing
Source: PLoS Negl Trop Dis. 2020 Nov 9;14(11):e0008831. doi: 10.1371/journal.pntd.0008831 (PMC7676723; doi:10.1371/journal.pntd.0008831)
Supplement: S2 Fig — (PDF) [file pntd.0008831.s002.pdf]

Supplementary Figure 2. Geometric mean ratio of half-lives, averaged across all four surfaces.

Effect	Surfaces Compared		Estimate	p-value
Surface	$1 U.S.A. Bank Note	$20 U.S.A. Bank Note	1.17	0.5254
Surface	$1 U.S.A. Bank Note	Clothing	1.44	0.1621
Surface	$1 U.S.A. Bank Note	Skin	0.55	**0.0400**
Surface	$20 U.S.A. Bank Note	Clothing	1.22	0.3242
Surface	$20 U.S.A. Bank Note	Skin	0.47	**0.0075**
Surface	Clothing	Skin	0.38	**0.0015**
